# Effect of Supplementation of Herd Diet with Olive Cake on the Composition Profile of Milk and on the Composition, Quality and Sensory Profile of Cheeses Made Therefrom

**DOI:** 10.3390/ani10060977

**Published:** 2020-06-04

**Authors:** Biagina Chiofalo, Ambra Rita Di Rosa, Vittorio Lo Presti, Vincenzo Chiofalo, Luigi Liotta

**Affiliations:** 1 Department of Veterinary Sciences, University of Messina, 98168 Messina, Italy; dirosaa@unime.it (A.R.D.R.); vittorio.lopresti@unime.it (V.L.P.); luigi.liotta@unime.it (L.L.); 2 Consortium Research of Meat and Agrifood, 98168 Messina, Italy; 3 Department of Chemical, Biological, Pharmaceutical and Environmental Sciences, University of Messina, 98166 Messina, Italy; vincenzo.chiofalo@unime.it

**Keywords:** olive pomace, dairy cow, milk, cheese, chemical composition, fatty acid profile, sensory profile

## Abstract

**Simple Summary:**

Replacing conventional feed with waste biomass produced by crop-industrial processes can be a good practice for the sustainability of crop-livestock systems and an interesting solution for their disposal—as long as they maintain the quality of products. Considering the relationship between feeding management and qualitative profile in products of animal origin, the aim of our study was to assess the effect of dietary olive cake supplementation on nutritional quality, fatty acid composition, volatile and sensory profile of milk and the cheese produced by dairy cows. The experiment was carried out on eighty-four healthy dairy Friesian cows, divided into two homogenous groups. Animals were fed with a conventional diet (CTR group) and a conventional diet supplemented with dried olive cake (OC group). Data showed that olive cake utilization in the diet of lactating dairy cows may modify the quality of dairy products. The increased unsaturated fatty acids (oleic acid, vaccenic acid and CLA) and decreased SFA (short- and medium-chain fatty acids) suggest a positive role of olive cake in improving the nutritional and nutraceutical properties of the cheese. Moreover, the olive cake affected not only the volatile profile of the cheese, but also its appearance, smell and taste, which are associated with a higher score of acceptance.

**Abstract:**

Aim of the present study was to assess the effect of dietary dried partially destoned olive cake supplement on nutritional quality and sensory profile of milk and cheese produced by dairy cows. The experiment was carried out on eighty-four healthy dairy Friesian cows divided into two homogenous groups. The control group (CTR) received a conventional diet, whereas the experimental group (OC) received a conventional diet supplemented with olive cake as 15% of DM. The trial lasted five months. Monthly, on individual milk samples, yield and physical-chemical parameters were determined. Milk was used for the artisanal cheese production. On 10 samples of cheese for each group, physical-chemical and fatty acid profile were determined. Electronic nose analysis and sensory evaluation were performed. Data were analyzed by ANOVA. The diet affected (*p* < 0.05) the milk yield, exclusively in September. Yield and quality of cheese of OC group after 60 d of ripening showed higher (*p* < 0.05) yield, moisture and fat content, lower (*p* < 0.05) pH, protein, salt and ash content, higher (*p* < 0.01) MUFA and PUFA and CLA content, lower (*p* < 0.05) SFA, higher (*p* < 0.01) UFA/SFA and hypocholesterolemic/hypercholesterolemic ratios, better (*p* < 0.01) atherogenic and thrombogenic indices. Data show dietary olive cake supplementation in lactating dairy cows improves nutritional and nutraceutical properties of cheese, volatile profile and level of assessors’ acceptance.

## 1. Introduction

The Mediterranean area is home to the principal olive production region that is about 76% of the world’s olive oil production. Italy represents the second country for olive oil production with a production of more than 429,000 tons per year [1]. Olive fruits (drupes) contain about 50% of water and another 50% of oil and dry matter, in an equal part [2]. Therefore, a large quantity of the organic matter represented by the vegetation water (wastewater) and by the pomace (crushed stone, skin and pulp with a nonnegligible presence of oil), after the oil extraction, is produced. The olive cake is characterized by a low crude protein content (<10% of DM), a high crude fiber content (up to 50% of DM) and up to 15% of ether extract (mainly oil) which is composed, principally, by the monounsaturated fatty acids (mainly C18:1 cis-9, oleic acid) [3]. The use of olive cake as animal feed represents a sustainable alternative to the disposal of biodegradable organic matter reducing the costs associated with animal nutrition and allowing a rational utilization of this residual biomass. In this context, getting rid of by-products must not compromise the quality of products.

It is well-known that the diet, whether or not mediated by the rumen metabolism, can modify the fatty quality of dairy products. Few studies showed a significant modification in milk quality (e.g., fatty acid composition and sensory profile) in relation to the olive cake supplementation. Several trials tested a dietary olive cake supplement in ruminants, and they reported no modification in the milk yield [4,5,6] and in the animal performance [7,8,9]. An experiment carried out on ewes fed with a dietary integration of partly stoned olive cake showed an increase in the UFA/SFA ratio and a decrease in the atherogenic and thrombogenic indices of milk [10]. These observations were confirmed by Abbeddou et al. [11] and Vargas-Bello-Pérez et al. [12] in dairy ewes supplemented with the olive pomace. Functionality and risks to human health posed by foods of animal origin are an increasing concern. Fats from ruminant products (milk, cheese, meat) are characterized by a category of fatty acids that are positively correlated with human health [13,14]. Evidence supports the relationship between feeding management and the aromatic profile in products of animal origin. Therefore, it is important to improve the understanding of the relationship between feeding management strategies and nutritional and nutraceutical properties of dairy products.

The use of olive cake byproduct could represent an opportunity to improve the sustainability of crop-livestock systems, maintaining the sensory and health properties of animal origin products. Bendal et al. [15] observed a significant modification in the milk aromatic profile of cows fed with a conventional diet or on pasture. Significant modifications of the milk aromatic profile were observed in cows [16] and ewes [17] fed diets containing high levels of unsaturated fatty acids. However, Caputo et al. [18] reported no effects on the milk and on the cheese aromatic profile of cows fed with a dietary olive pomace supplement.

The cheese flavor and the flavor quality are critical parameters for the acceptability of dairy products. Substantially, the flavor is a sensory perception and can be analytically described by a descriptive sensory analysis, and the product can be evaluated by a group of trained assessors who act as an instrument [19]. The volatile fraction of cheese contains greater than 200 compounds. Not all the volatile compounds contribute to flavor, however, and the stimulus responsible for cheese flavor (odor, aroma and taste sensations) is widely believed to be a type-specific, balance and concentration of numerous aromatic and non-volatile sapid compounds. Therefore, it is expected that headspace procedures, such as the electronic nose are at best sensitive to the volatile components of odors when physiological factors such as flavor release and perception during consumption are considered. For this reason, in recent years, the electronic nose, an instrument with an array of sensors for volatile compound detection, has been widely used to design a new sensory fingerprint model of food of animal origin and offers a potential alternative for discrimination of multiple samples [20,21].

The present study aimed to assess the effect of the dietary dried partially destoned olive cake *cv. Menfi* (DOC) supplement on the composition profile of milk on the chemical composition, quality and sensory profile of cheeses made therefrom.

## 2. Materials and Methods

### 2.1. Ethical Statement

All the procedures followed in this study were in agreement with the European guidelines for the care and use of animals in research [22]. The research received institutional approval by the Regional Department of Agriculture, Rural Development and Mediterranean Fisheries of the Sicilian Region (Dipartimento Regionale Agricoltura—Assessorato Regionale dell’Agricoltura, dello Sviluppo Rurale e della Pesca Mediterranea—Regione Siciliana), Italy, n. 94750023262, prot. 110180.

### 2.2. Animals and Diets

The experiment was performed into a commercial dairy farm located in the Sicilian region (Italy). Eighty-four healthy dairy Friesian cows were divided into two groups (42 animals each), homogenous for the weight (550 kg), distance from calving (80–90 d) and milk production (26 kg/d), fed a conventional diet (20 kg of DM/head per day as TMR) with concentrate (Table 1) and meadow hay (CP: 110.9 g/kg of DM; EE: 25.0 g/kg of DM; NDF: 521.9 g/kg of DM; NE_L_ 0.65 UFL/kg of DM). The control group (CTR) received a concentrate without any olive cake supplements, whereas the experimental group (OC) received a concentrate supplemented with the olive cake (DOC) as 15% on a DM (Table 1). Feed was collected at the beginning, middle and end of the trial. The total amount collected at each time and for each group was mixed, and a final sample was taken. After drying, the olive pomace was stored in boxes and sampled from the top, middle and bottom. The total amount collected at each time was pooled, and a final sample was taken. Samples of feed were thereafter analyzed for chemical composition. Ingredients and chemical composition of the CTR and OC diets and of the DOC are reported in Table 1 and Table 2. During the 2 weeks of the adaptation period, the DOC was increased daily up to 2 kg of DM; this was achieved on Day 14, which was considered time zero.

### 2.3. Sampling

The experimental period lasted from May to September 2019. Monthly, after the morning complete mechanical milking, the individual daily milk production was registered from each milking bucket and 3 milk samples, of 100 mL each one, were taken from each milking bucket. The milk samples, for a total of 252 milk samples (84 animals × 3 samplings), were transported under refrigerated conditions (4 ± 2 °C) and subjected to the physical-chemical analysis.

**Figure 1:**
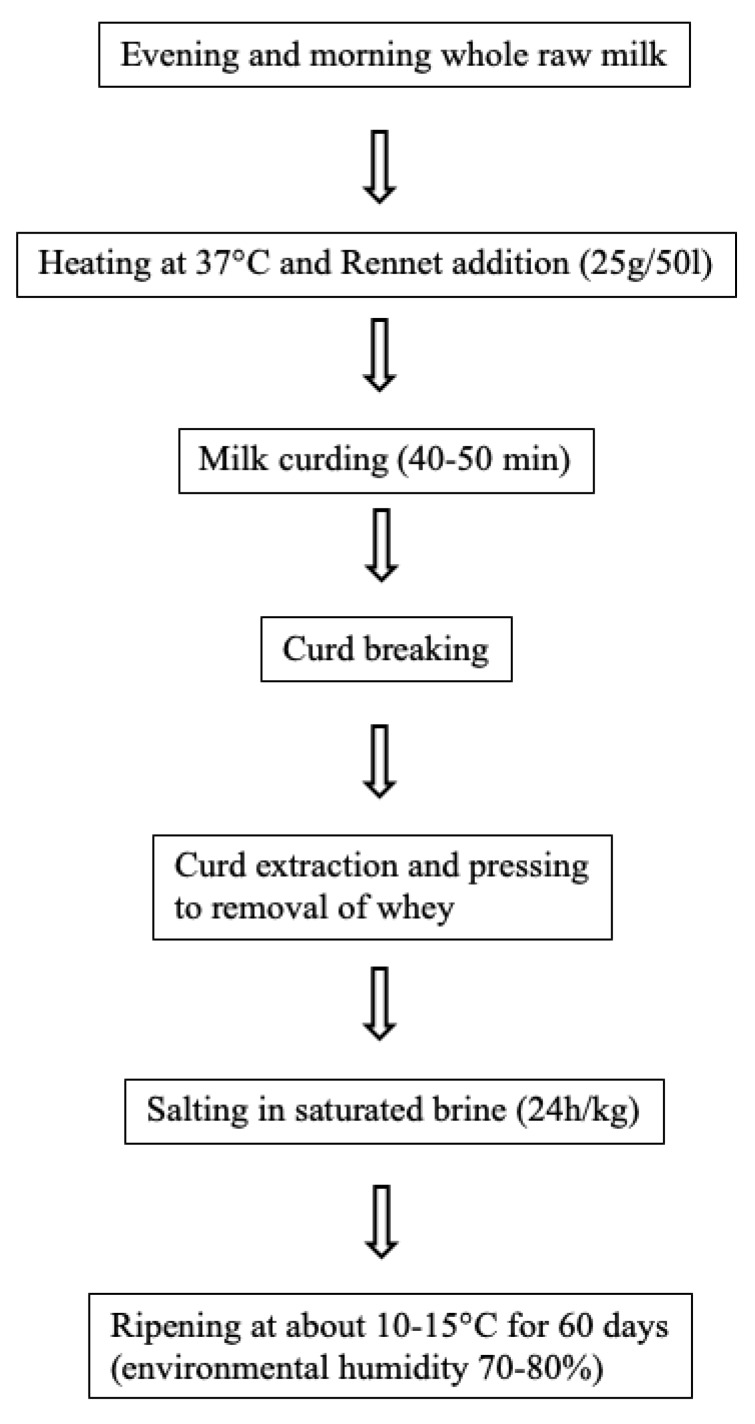
Flowchart of cheese production.

Daily, the milk of the trial was collected, separately (CTR and OC groups), into the tanks of the farm and used for the production of artisanal cheese, called “Canestrato”, by using a traditional practice (Figure 1). The rennet was added to the milk and the clotting occurs as a combined effect of the rennet and the natural microflora of the milk which acts as starter cultures. Cheeses were seasoned for 60 days in a ripening room of the factory, at the temperature of 10–15 °C and at the humidity of 70%–80%. Ten ripened cheese wheels, for each group, produced by using the milking of September were collected, vacuum packaged and stored under refrigerated conditions (4 ± 2 °C); for each ripened cheese, three portions were sampled and subjected to the physical-chemical analysis, for a total of 60 samples analyzed (10 cheese wheels × 2 groups × 3 samplings).

The measurement of the yield (expressed in kg) was recorded on each fresh cheese obtained from 100 kg of milk. The measurement was repeated ten times (one for each cheese of the two groups).

### 2.4. Physical-chemical Analysis of Feed, Milk and Cheese

Samples of TMR and DOC were analyzed for DM (method 930.15 1999), CP (method 2001.11 2005), EE (method 920.39 1920) and ash (method 942.05 1942) according to AOAC International [24]. The NDF and ADF were determined using the method reported by Goering and Van Soest [25].

Individual milk samples (250 mL) were analyzed for the fat, protein, casein, lactose and urea content by using a Fourier transform infrared (Milkoscan FT2, FOSS, Hillerød, Denmark) and the somatic cell count was determined using a Fossomatic^TM^ FC (FOSS, Hillerød, Denmark). Aliquots (100 g) of the cheese ripened of a mix of sections of both core and the surface cheese were analyzed for the moisture, fat, protein and salt content, using a near infrared spectroscopy in transmittance (FoodScan^TM^ Dairy Analyzer; FOSS, Hillerød, Denmark). In addition, the pH value of milk and cheese samples was determined by a pH meter (H19017, Microprocessor, Hanna Instruments, Ronchi di Villafranca Padovana, Padova, Italy).

### 2.5. Fatty Acids of Feed and Cheese

The fatty acid methyl esters (FAME) of feed and cheese were analyzed by a GC–FID. For the chromatographic analyses, on 15 mg of lipids of feed and cheese, the fatty acids methyl esters were prepared [26] by using a solution of sulfuric acid/methanol (1:9, *v/v*) and submitted to a HRGC analysis.

For feed, a gas chromatograph with a FID detector (Agilent Technologies 6890 N, Palo Alto, CA, USA) equipped with an Omegawax 250 column (Supelco, Bellefonte, PA, USA; 30 m × 0.25 mm i.d., 0.25 μm film thickness) was used. The column temperature was programmed: an initial isotherm of 100 °C (5 min), an increment of 4 °C/min and a final isotherm of 240 °C (20 min). The temperature of the injector and detector was 250 °C. The injection volume was 0.5 μL. The carrier gas used was helium (1 mL/min), and the split ratio was 1:50.

For the cheese, a gas chromatograph with a FID detector (Agilent Technologies 6890 N, Palo Alto, CA, USA) equipped with a SP-2560 fused silica capillary column (100 m × 0.25 mm i.d. × 0.2 µm film thickness, Supelco, Inc., Bellefonte, PA, USA.) was used. Helium was the carrier gas, 1 mL/min. The volume of the injection was 1 µl and the split ratio 1:50. The column temperature was programmed: an initial isotherm 70 °C (2 min), an increment of 15 °C/min to 155 °C (held for 25 min), then an increment of 3 °C/min and a final isotherm of 215 °C (8 min), following the procedure described by Tudisco et al. [27].

The fatty acids of the feed and cheese were identified by comparing the relative retention times of FAME peaks from samples containing standards from Supelco (mix 37 FAMEs, Supelco, Bellefonte, PA, USA). Chromatogram peak areas were acquired and calculated using a Chemstation software (Agilent, Santa Clara, CA, United States). The concentration of each fatty acid was expressed as g/100 g, considering 100 g as the summation of the areas of all the FAME identified. For each sample, the chromatographic analysis was repeated three times.

The amount of each fatty acid was used to calculate the indices of atherogenicity (AI) and thrombogenicity (TI), as proposed by Ulbricht and Southgate [28] and the hypocholesterolemic/hypercholesterolemic ratio (HH), as suggested by Santos-Silva et al. [29]:

(1) $$AI=\frac{C12:0+4\;\left(\;C14:\;0\right)+\;C16:0}{\Sigma \;\mathrm{MUFA}\;+\;\Sigma \;n-6\mathrm{PUFA}+\;\Sigma \;n-3\mathrm{PUFA}}$$

(2) $$TI=\frac{C14:0+C16:0+C18:0}{\;\left(\;0.5\times \Sigma \;MUFA\right)+\left(0.5\times \Sigma \;n-6\mathrm{PUFA}\right)+\left(3\times \Sigma \;n-3\mathrm{PUFA}\right)+\left(\Sigma \;n-3\mathrm{PUFA}/\Sigma \;n-6\mathrm{PUFA}\right)}$$

(3) $$HH=\frac{C18:1n-9+C18:2n-6+C20:4n-6+C18:3n-3+C20:5n-3+C22:5n-3+C22:6n-3}{C14:0+C16:0}$$

### 2.6. E-nose Analysis of Cheese

The odor fingerprint analysis on five cheese wheels per group was evaluated using an e-nose aFOX-4000 (Alpha M.O.S., Toulouse, France), with 18 metal oxide semiconductor (MOS) sensors and an autosampler (A HS100). The cheese wheels were thawed and a portion along the central part of each was sampled and chopped into small portions with a clean kitchen knife. Two grams (±0.10) of samples were put into 10-mL-headspace vials and sealed then incubated at 35 °C for 30 min, at 500 rpm under intermittent shake. After the incubation, 500 μL of headspace was withdrawn by a gas-tight 5-mL syringe kept at 50 °C and pumped by the carrier gas into sensor chambers at a flow rate of 150 mL/min [30]. The change in sensor resistance was collected for 120 sec. The next injection was pumped after 18 min for baseline recovery. The eighteen signal responses (one for each sensor) represent a sample in the data matrix used for the statistical analysis.

### 2.7. Sensory Analysis of Cheese

A sensory acceptance test was carried out by a panel of 20 cheese consumers (14 males and 6 females), ranging from 20 to 50 years old, were recruited at random from the Department of Veterinary Sciences to take part in the sensory analysis. The criterion for the selection of panelists was the cheese consumption, at least, once per week [31,32]. Assessors were instructed to evaluate the cheese for the degree of liking of the “appearance”, “color”, “texture”, “taste”, “odor” and “overall acceptance” using a 7-point hedonic scale (0 = poor, 6 = excellent) [33,34]. The sample was removed from the refrigerator, cut into portions (about 1.5 × 1.5 × 1.5 cm) and served at 22 °C to the assessors with randomly assigned three-digit codes in a randomized order. Some water and a cracker were provided between each sample.

### 2.8. Statistical Analysis

For milk quality traits, data were analyzed with a mixed model considering diet and milk sampling as experimental factors, and individual cow as random factor, (repeated measure design—GLM procedure of SAS) [35]:

Y_i,j,k_ = µ + D_i_ + P_j_ + (D × P)_i,j_ + α_k_ + ε_i,j,k_ (4)

where Y_i,j,k_ are observations, µ is the overall mean, D_i_ is the fixed effect of diet i (i = 2), P_j_ is the fixed effect of period j (j = 5 for milk yield and quality), (D × P)_ij_ is the interaction between dietary treatment and period, α_k_ is the random effect of animal k (k = 84) and ε_i,j,k_ is the random residual. Comparisons between treatment means were performed using the Tukey’s test. Differences were considered significant at *p* < 0.05.

For cheese quality traits (gross composition and fatty acids), data were analyzed by a 1-way ANOVA [35], considering diet as the variable. Separation of means was assessed by Tukey’s test, and differences were considered significant if *p* < 0.05. Results are shown as least squares means ± standard error of the mean.

Data of the aromatic e-nose profile were analyzed through Alpha Soft V12.4 (Alpha-MOS, Toulouse, France) performed a principal components analysis (PCA), used like first and exploratory analysis to verify the discrimination capability between the two groups of cheeses. The effectiveness of discrimination was assessed on the discrimination index (DI), which reach a maximum value of 100 when the groups are completely resolved [36].

## 3. Results

### 3.1. Dried Olive Cake (DOC) and Diet

The fatty acid profile of the DOC and diets is shown in Table 2 and Table 3. The most abundant fatty acid in the DOC was oleic acid (C18:1n9). The palmitic (C16:0) and the linoleic (C18:2n6) acids were the other fatty acids that were also present, whereas the stearic (C18:0) and linolenic (C18:3n3) acids were the fatty acids less present in the DOC (Table 2).

The OC diet showed a higher content of the oleic acid and a lower content of the linoleic and linolenic acid compared with those of the CTR diet. Consequently, the monounsaturated fatty acid class showed a higher content whereas, polyunsaturated fatty acid class a lower content in the OC diet than those of the CTR diet (Table 3).

### 3.2. Yield and Nutritional Composition of Milk and Cheese

Table 4 shows the results from ANOVA for the milk yield and quality traits (F-value and significance) for fixed effects (diet, period and their interaction). Table 5 shows the effect of olive cake supplement on milk yield and composition.

Significant differences among periods (Table 4) were not reported in Table 5 since they depend on the lactation stages and the average monthly milk yield in lactation (mean ±standard deviation).

The diet affected significantly (*p* < 0.05) the milk yield (Table 4), exclusively in September, when the OC group showed a significant (*p* < 0.05) higher milk yield than that of the CTR group (Table 5). No significant differences were observed for milk quality traits in relation to the diet (Table 4 and Table 5).

As regards the effect of the period (Table 4), a significant (*p* < 0.05) difference was observed for the milk yield and the fat and urea content, as expected in relation to the stage of lactation. The values of milk urea content were within the recommended range (23–35 mg/100 mL) [37] with the exception of urea content in May and July (Table 5), when the contents were under the minimum values of the recommended range.

The interaction of diet x period did not influence the milk yield and the quality traits (Table 4).

The yield of fresh cheeses showed no significant (*p* = 0.12) differences between the groups (11.5 kg vs. 11.2 kg, respectively for the OC group and CTR group).

The chemical composition of cheeses after 60 d of ripening is reported in Table 6. The cheese produced from milk of dairy cows that received the DOC supplementation showed a chemical composition similar to that of the control group fed with a conventional diet.

### 3.3. Cheese Fatty Acids Composition

Data regarding the fatty acid profile recorded in cheeses at the beginning of the ripening time were not reported whereas, only data for cheeses after 60 days of ripening (which corresponds to the marketing time) are shown (Table 7 and Table 8). The DOC inclusion modified the fatty acid profile of cheeses. Significant changes involved the butyric (*p* < 0.05), caproic (*p* < 0.05), caprylic (*p* < 0.05), palmitic (*p* < 0.05), stearic (*p* < 0.05), oleic (*p* < 0.01), *trans* isomers of oleic acid (vaccenic acid; *p* < 0.01), linoleic (*p* < 0.01) and CLA isomers (C18:2 *cis*-9, *trans*-11 and C18:2 *trans*-10, *cis*-12, *p* < 0.01 and *p* < 0.05, respectively) (Table 7).

The DOC supplement produced a significant (*p* < 0.01) increase in the MUFA and PUFA, and a significant (*p* < 0.05) decrease in the SFA (Table 8). The UFA/SFA and the hypocholesterolemic/hypercholesterolemic ratios were significant (p < 0.01) higher for the cheese of OC group than those of the CTR. As a consequence, the atherogenic and thrombogenic indices were reduced significantly (*p* < 0.01) in OC cheeses. Finally, the DOC supplement was associated with a significant (*p* < 0.01) increased CLA content in cheeses (Table 8).

### 3.4. Electronic Nose Aromatic Profile of Cheese

**Figure 2:**
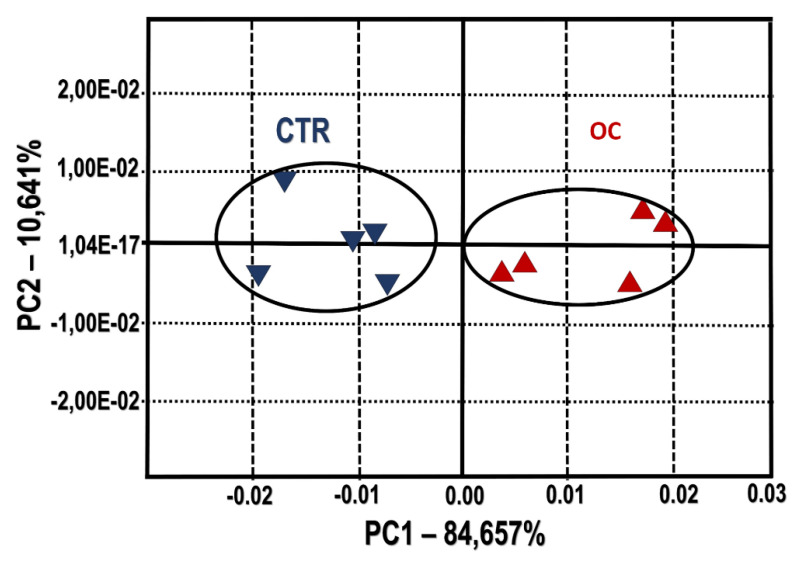
Principal Components Analysis (PCA) plot of cheese samples. CTR group: five cheese wheels after 60 days of ripening obtained from the milk of dairy cows fed concentrate without olive cake supplements. OC group: five cheese wheels after 60 days of ripening obtained from the milk of dairy cows fed concentrate with olive cake supplements. Discrimination index = 59. The two principal components (PC1 and PC2) are responsible for 95.3% of the variation showed by sensor’s signals used as loadings.

The odor map done with all sensors involved, described the two principal components that accounted for more than 90% of the total explained variance (Figure 2).

**Figure 3:**
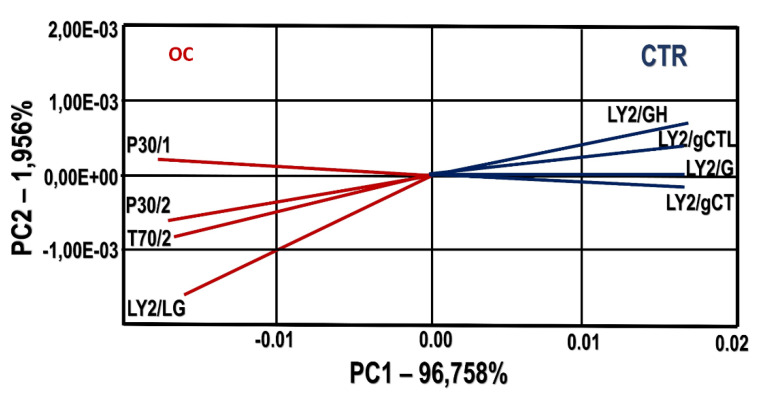
Principal Components Analysis (PCA) with the loading of selected sensors for the cheese samples. CTR group: five cheese wheels after 60 days of ripening obtained from the milk of dairy cows fed concentrate without olive cake supplements. OC group: five cheese wheels after 60 days of ripening obtained from the milk of dairy cows fed concentrate with olive cake supplements. LY2/GH, LY2/gCTL, LY2/gCT, LY2/G are the selected sensors involved for CTR group and LY2/LG, P30/1, T70/2, P30/2 are the selected sensors involved for OC group. Discrimination index = 74. The two principal components (PC1 and PC2) are responsible for 98.71% of the variation showed by sensor’s signals used as loadings. The loading length of a trait measures the magnitude of its effect (positive or negative).

The plot shows a significant separation of samples, mainly observed on the *x*-axis, by the first component (84.66%). The discrimination index obtains a positive value of 59, because there is no intersection on the surface between groups and no dispersion in each one, thereby as a preliminary analysis, the discrimination of both groups is possible. To obtain the best variables for the analysis, a combination of sensors whose response provided the highest discrimination power was selected. In Figure 3 the PCA plot is built with eight sensors. Figure 3 shows a value for the discrimination index that changed from 59 to 74. The sensors involved four for each group were four: LY2/GH, LY2/gCTL, LY2/gCT, LY2/G for CTR group and LY2/LG, P30/1, T70/2, P30/2 for OC group.

### 3.5. Sensory Profile of Cheese

**Figure 4:**
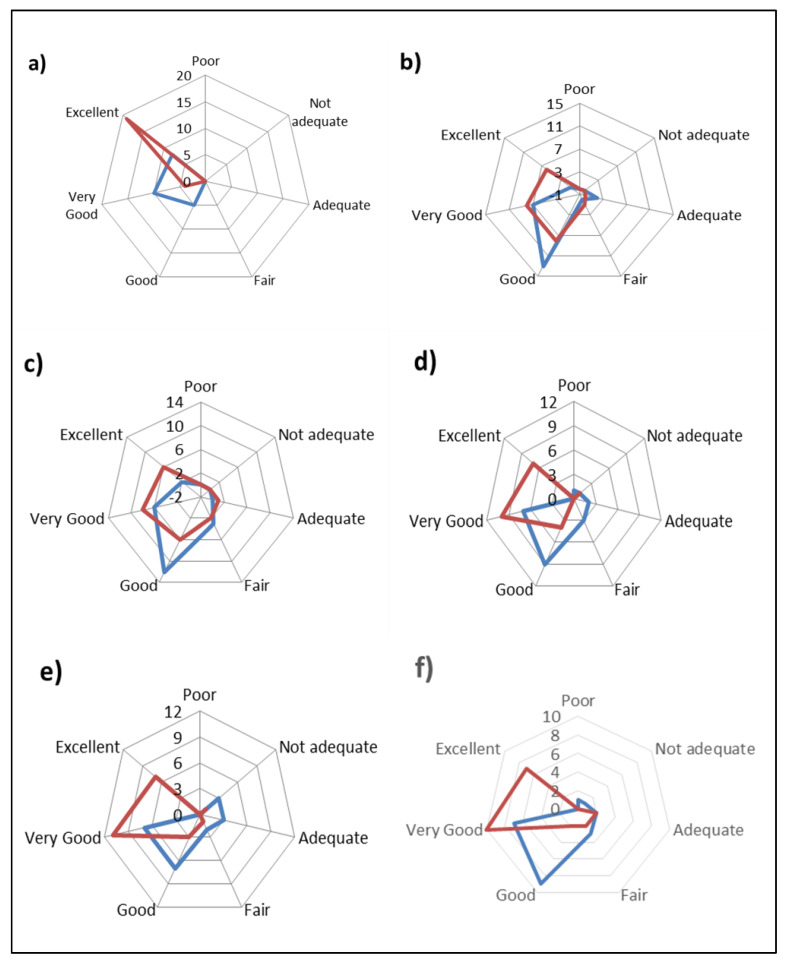
Consumer evaluation of cheeses from two groups CTR cheese (blue) and OC cheese (red) for: (**a**) appearance; (**b**) color; (**c**) texture; (**d**) smell; (**e**) taste; (**f**) overall acceptance. CTR group: five cheese wheels after 60 days of ripening obtained from the milk of dairy cows fed concentrate without olive cake supplements. OC group: five cheese wheels after 60 days of ripening obtained from the milk of dairy cows fed concentrate with olive cake supplements.

The results of the sensory acceptance test are shown in Figure 4. All the characteristics evaluated, “appearance”, “color”, “texture”, “smell” and “taste”, cheese from the milk of animals fed with olive cake showed the highest scores. The smell and the taste profile turned out to be interesting, highlighting not only a clear differentiation between the cheese of the two groups, but also a tendency to prefer the cheese of the OC group. As a complement to the descriptive analysis, cheeses made with the dietary olive cake supplement had a higher level of acceptance.

## 4. Discussion

Data showed no significant differences in the milk yield and in the chemical composition in relation to the olive cake supplement, according to other researchers who studied the effect of dietary dried stoned olive pomace integration (about 15% on a DM) in cows [5], buffalo [4] and ewes [11].

The yield of fresh cheeses and the chemical profile of cheeses after 60 days of ripening were not affected by the DOC dietary integration; these results are in agreement with the observations of Abbeddou et al. [38] and Branciari et al. [39] in dairy sheep.

As regards the fatty acid profile, the dietary DOC supplement reduced short- and medium-chain (C4:0, C6:0, C8:0 and C16:0) fatty acids. Short- and medium-chain fatty acids are synthetized completely “de novo” and partially by the mammary gland starting from the acetate and beta-hydroxybutyrate, respectively, produced in the rumen [40]. Other studies report that supplement with long-chain fatty acids negatively affects “de novo” synthesis of the short- and medium-chain fatty acids in the mammary gland [41]. Medium-chain fatty acids are responsible for increasing the concentration of low-density lipoprotein cholesterol in the blood when it is not associated with the correct level of linoleic acid [42].

The high oleic acid content observed in cheeses of the OC group is related to the high level of oleic acid into the diet and, probably, to the desaturation of the stearic acid occurring in the mammary gland by the delta9-desaturase [43], encoded by the stearoyl coenzyme A desaturase gene [44], as reported by Chiofalo et al. [10] and Vargas-Bello-Pérez et al. [12] in dairy sheep and by Terramoccia et al. [4] in buffalo fed dietary olive pomace supplement. Moreover, the oleic acid content could also be related by a partial lack of the rumen bio-hydrogenation of the oleic acid (converted into the stearic acid). Probably, the higher NDF diet’s content of the OC than that of CTR group caused an increase of the rumen transit speed and a partial inactivity of rumen specific microorganisms, involved in the stearic synthesis too [10].

Previous studies have investigated the effect of olive cake on the rumen microbial community, in particular *Butyrivibrio* genus and *Butyrivibrio proteoclasticus*, which are responsible for the biohydrogenation of unsaturated fatty acids and the reduction of oleic in stearic acid, respectively. Olive cake negatively affected the *Butyrivibrio* genus and *B. proteoclasticus* activity and slowed the hydrogenation of oleic and linoleic intermediates in ewes [45], suggesting a partial escape of the biohydrogenation intermediates from the rumen [46]. Our results are consistent with previous studies that highlighted that dietary supplementation with ingredients containing high oil content produced a higher level of C18:0, C18:1, C18:1 trans-11 and of the C18:2 cis-9,trans-11 in goats [47] and cows [48].

The high level of stearic acid in the cheese of the OC group testify a microbial biohydrogenation of the dietary unsaturated fatty acids (MUFA and PUFA) as reported by Mosley et al. [49] and as observed by Gómez-Cortés et al. [50] and Moate et al. [51] in products obtained by dairy animals fed with ingredients containing high levels of unsaturated C18 fatty acids. Nevertheless, the increase of the CLA isomers could be due to the secretion of biohydrogenation intermediates (vaccenic acid—C18:1 trans 11, in particular) caused by the dried olive cake supplement. This synthesis in milk (mainly of vaccenic and rumenic) is regulated by the stearoyl coenzyme A desaturase activity in the mammary tissue.

The explanation of these observations could be related to the double origin of the rumenic acid that is, partially synthesized in the rumen by the biohydrogenation of the linoleic acid, responsible for the production of a small proportion of the CLA isomers secreted in milk [52] and, partially, synthesized in the mammary gland starting from the vaccenic acid [40], responsible for about 60% of the rumenic acid secretion in cow milk [53,54]. The CLA isomers major represented in dairy products are the rumenic acid and the CLA trans-10, cis-12. In our study, these fatty acids showed a significantly higher level in cheeses of the OC group than those of the CTR group.

Recently, studies have highlighted their interesting role in the functionality of the mammary gland, mediated by its antioxidant activity. Basiricò et al. [55] report the role of CLA isomers in the protection from the lipoperoxidation of bovine mammary epithelial cells. Moreover, CLA isomers mitigate the level of the reactive oxygen species better than other fatty acids [56].

From a nutritional point of view, ruminant products are the primary dietary source of the CLA isomers for humans. Their beneficial effects are due to the potential activity in slowing the development of atherosclerosis [57], reducing the accumulation of the body fat [58], improving the bone mineralization [59], modulating the immune system [60] and to their influence on the glucose and lipid metabolism [61].

The SFA, MUFA and PUFA content in cheeses was influenced by the dietary olive cake supplement as well as the UFA/SFA ratio, the atherogenic and thrombogenic indices and the ratio hypocholesterolemic/hypercholesterolemic fatty acids (HH), computed according to present knowledge of the effects of individual fatty acids on cholesterol metabolism [62,63]. The last dietary guidelines emphasize the health benefit of oleic acid on blood cholesterol and other related outcomes in humans, in fact, although the cholesterol-lowering response to polyunsaturated class is greater than that to the monounsaturated class, there has been some caution in recommending high polyunsaturated diets, because of potentially adverse health effects of their lipoperoxidation products [10]. Therefore, it could be argued that the dietary DOC supplement may influence the nutritional value and the health functionality of animal products [64] and could represent a valid option to reduce the feeding cost and the environmental impact related to the food production [65,66].

Regarding the application of the e-nose system on the volatile profile of cheeses, the results allow to distinguish four sensors, three LY2 and one *P-*type metal oxide semiconductor. LY2/LG and P30/2 characterize the odor of the OC group, with the selectivity for oxidizing gases and alcohol compounds. The odor difference between samples could be due to the different fatty acid composition of cheeses, since it is known a strong link of the milk fat and the development of the flavor in the cheese [20,67]. LY2/GH and LY2/gCTL were significant in defining the odor of the CTR group. These sensors have response selectivity for amines and Sulphur derivatives, typically found in the cheese [68]. Both compounds are generally produced for the catabolism of free amino acids contributing to the odor profile. Sulphur and amine compounds are reported to be characterized by a low odor threshold perception, thus markedly influencing the cheese flavor and they make a significant contribution to the cheese typical flavor [67,68,69]; the different concentration of these compounds could vary in relation to the animal diet [70,71]. This in agreement with Castellani et al. (2018) [72] that affirmed that in dairy cows, dietary supplementation of dried olive pomace may affect proteolytic volatile compounds in milk and cheese. The ability of the electronic nose to respond to physicochemical characteristics contribute to the discrimination of the volatile compounds of the cheese.

During the consumer acceptance test, the assessors have shown a great appreciation for all the evaluated characteristics of cheeses, even if the content of some volatile compounds was modified.

## 5. Conclusions

Data show that the dried partially destoned olive cake added into the diet of lactating dairy cows modified the quality of dairy products. The increase of unsaturated fatty acids (oleic acid, vaccenic acid and CLA) and the decrease of saturated fatty acids suggests a positive role of olive cake on the nutritional and nutraceutical properties of cheese. The aromatic profile of cheese was also modified by the DOC integration and the e-nose was able to discriminate the volatile profile of cheese from different groups. Thereby, the use of e-nose could represent as an effective tool to recognize a product obtained from animals fed with alternative eco-friendly resources and addressed to minimize the environmental impact of the food industry. In addition, cheeses made from the milk of animals fed with an olive cake supplement had a higher level of consumer acceptance.

The substitution of conventional feed with waste biomasses produced by agroindustrial processes (e.g., olive cake) represents a good practice for increasing the sustainability of dairy products. Finally, the valorization of the dried partially destoned olive cake as animal feed is an interesting solution to its disposal, especially in areas where these by-products are produced in high amounts.

## Figures and Tables

**Table 1:** Ingredients and nutritional characteristics of the concentrates.

Item	Control Group (CTR)	Olive Cake Group (OC)
Ingredient, kg of DM		
Corn meal	390	375
Soybean meal (48% CP)	190	175
Barley meal Wheat middling	150 120	115 115
Sunflower meal	60.0	20.0
Dried Olive Cake *cv. Menfi*		150
Beet pulp	60.0	20
Calcium carbonate	7.0	7.0
Na bicarbonate	5.0	5.0
Na chloride	5.0	5.0
P dicalcium	4.0	4.0
Mg oxide	3.0	3.0
Na propionate	1.0	1.0
Vitamin premix ^§^	5.0	5.0
Chemical composition, g/kg of DM		
Moisture	106	113
Starch	396.5	403.8
Crude protein	168	170
Ether extract	45.7	44.2
Neutral detergent fiber	217.6	239.4
Acid detergent fiber	89.2	131.8
Acid detergent lignin	21.3	48.3
Ash	66.8	73.0
Calculated nutrient composition		
NE_L_, milk UFL/kg of DM	1.08	1.09
Ca	8.6	8.5
P	1.9	2.7

CTR group: dairy cows fed concentrate without olive cake supplements. OC group: dairy cows fed concentrate with olive cake supplements. DM: Dry Matter. CP: Crude Protein. Na: Sodium. P: Phosphorus. Mg: Manganese. ^§^ Providing per kg of diet: 32,000 U vitamin A, 3200 U vitamin D_3_, 120 mg vitamin E, 8 mg vitamin B_1_, 1.6 mg vitamin B_2_, 0.016 mg vitamin B_12_, 400 mg niacin, 4 mg pantothenic acid, 400 mg choline chloride. NE_L_: net energy lactation. Milk production efficiency was calculated based on the net energy system [23], where one milk forage unit (UFL) of energy is defined as the net energy content of 1 kg of standard barley for milk production, equivalent to 1700 kcal.

**Table 2:** Chemical and acidic composition of the dried olive cake used in the trial.

Dried Olive Cake	
**Chemical composition**	**(g/kg as fed)**
Moisture	43.9
Crude protein	86.3
Ether extract	303.4
Neutral detergent fiber	493.7
Acid detergent fiber	393.9
Acid detergent lignin	230.6
Ash	40.9
Starch	14.8
**Fatty acids**	**(g/100 g of FAME) ^#^**
C14:0	1.76
C16:0	14.43
C18:0	3.52
C18:1	67.18
C18:2	8.39
C18:3	0.52

^#^ Concentration of fatty acid is expressed as g/100 g, considering 100 g the sum of the areas of all fatty acid methyl esters (FAME) identified. C14:0 = Myristic acid. C16:0 = Palmitic acid. C18:0 = Stearic acid. C18:1 = Oleic acid. C18:2 = Linoleic acid. C18:3 = α-Linolenic acid.

**Table 3:** Fatty acids of nutritional interest, fatty acid classes (g/100 g FAME) ^#^ and unsaturated/saturated fatty acids ratio of the concentrates.

Diet	CTR	OC
Fatty acid profile		
C14:0	0.23	0.19
C16:0	15.80	14.92
C18:0	2.99	2.34
C18:1n9	22.57	42.58
C18:1n7	0.87	1.15
C18:2n6	52.23	34.16
C18:3n3	3.64	1.80
Fatty acid classes		
SFA	19.11	17.58
MUFA	23.84	45.27
PUFA	57.05	37.15
n-3 PUFA	4.25	2.40
n-6 UFA	52.16	34.46
Fatty acid ratio		
UFA/SFA	4.23	4.67

^#^ The concentration of fatty acid is expressed as g/100 g, considering 100 g the sum of the areas of all FAME identified. C14:0 = Myristic acid. C16:0 = Palmitic acid. C18:0 = Stearic acid. C18:1 = Oleic acid. C18:1n7 = cis-Vaccenic acid or cis-11-octadecenoic acid. C18:2 = Linoleic acid. C18:3 = α-Linolenic acid. SFA: Saturated fatty acids. MUFA: Monounsaturated fatty acids. PUFA: Polyunsaturated fatty acids.

**Table 4:** Results of the milk yield and quality traits from ANOVA (F-value and significance) for fixed effects (diet, period and their interaction) and RMS for random effects (animal and residuals).

Traits	F-Value of Fixed Effects			Random Effects (RMS)	
	Diet ^#^	Period ^†^	D × P ^‡^	Animal	Residuals
Yield (kg/day)	35.6 *	28.1 *	1.85	4.9	3.6
Fat (%)	0.8	8.5 *	1.55	0.6	0.4
Lactose (%)	0.9	0.9	1.80	1.1	0.9
Protein (%)	1.1	0.6	1.61	0.6	0.5
Casein (%)	0.9	0.7	0.98	0.4	0.2
Urea (mg/dL)	1.8	10.1 **	1.34	3.5	2.8
SCC ^§^ log_10_/mL	2.1	0.9	1.15	0.5	0.3

^#^ Diet: CTR group: milk produced by dairy cows fed concentrate without olive cake supplements; OC group: milk produced by dairy cows fed concentrate with olive cake supplements. ^†^ period: From May to September. ^‡^ D × P: interaction diet × period ^§^ Somatic Cell Count. * *p* < 0.05; ** *p* < 0.01.

**Table 5:** Effect of olive cake supplement on milk yield and composition (mean values ± SD).

Items	Yield (kg/day)		Fat (%)		Lactose (%)		Protein (%)		Casein (%)		Urea (mg/dL)		SCC ^§^ log_10_/mL	
	CTR	OC	CTR	OC	CTR	OC	CTR	OC	CTR	OC	CTR	OC	CTR	OC
May	23.8 ± 1.8	23.9 ± 1.6	2.57 ± 0.1	2.97 ± 0.3	4.59 ± 0.1	4.67 ± 0.1	2.69 ± 0.2	2.76 ± 0.3	2.28 ± 0.1	2.32 ± 0.1	18.33 ± 2.1	18.70 ± 2.1	2.51 ± 0.1	2.55 ± 0.1
June	23.3 ± 1.6	24.3 ± 1.1	2.83 ± 0.2	3.00 ± 0.2	4.61 ± 0.3	4.59 ± 0.2	2.59 ± 0.2	2.60 ± 0.2	2.23 ± 0.2	2.27 ± 0.2	24.50 ± 2.1	28.39 ± 2.1	2.54 ± 0.1	2.59 ± 0.1
July	24.6 ± 1.5	25.3 ± 1.5	3.30 ± 0.3	3.14 ± 0.4	4.72 ± 0.2	4.66 ± 0.1	2.86 ± 0.3	2.89 ± 0.3	2.49 ± 0.2	2.41 ± 0.2	22.22 ± 2.2	22.58 ± 2.3	2.56 ± 0.1	2.51 ± 0.1
August	23.6 ± 2.1	23.8 ± 1.4	3.07 ± 0.3	3.15 ± 0.4	4.76 ± 0.3	4.61 ± 0.2	2.94 ± 0.4	2.91 ± 0.3	2.47 ± 0.1	2.41 ± 0.2	23.78 ± 2.1	24.14 ± 2.9	2.54 ± 0.1	2.49 ± 0.1
September	23.7 ± 1.8 ^a^	25.1 ± 1.5 ^b^	3.46 ± 0.3	3.63 ± 0.3	4.57 ± 0.3	4.49 ± 0.4	3.11 ± 0.2	3.07 ± 0.2	2.47 ± 0.2	2.45 ± 0.2	24.45 ± 2.2	24.60 ± 2.1	2.59 ± 0.1	2.58 ± 0.1

CTR group: milk produced by dairy cows fed concentrate without olive cake supplements. OC group: milk produced by dairy cows fed concentrate with olive cake supplements. ^§^ Somatic Cell Count. ^a, b^ significant different for *p* < 0.05.

**Table 6:** Effect of olive cake supplement on gross composition of the cheese after 60 days of ripening.

Groups	No. Samples Analyzed ^†^	Cheese Parameters					
		pH	Moisture	Protein	Fat	Salt	Ash
CTR	10	5.48	38.61	27.84	27.31	2.04	3.84
OC	10	5.43	41.04	25.29	28.22	1.98	3.78
SEM		0.025	0.021	0.061	0.053	0.011	0.087
*p-*Value		0.14	0.08	0.07	0.11	0.35	0.22

CTR group: milk produced by dairy cows fed concentrate without olive cake supplements. OC group: milk produced by dairy cows fed concentrate with olive cake supplements. SEM: standard error of the mean. ^†^ Each sample of cheese was analyzed in triplicate. Values expressed in g/100 g edible part.

**Table 7:** Effect of olive cake supplement on cheese fatty acid profile (g/100 g) ^§^ after 60 days of ripening.

Fatty Acids	CTR	OC	SEM	*p*-Value
C 4:0	1.58	1.24	0.076	<0.05
C 6:0	1.69	1.27	0.209	<0.05
C 8:0	1.22	1.04	0.308	<0.05
C 10:0	2.98	2.72	0.035	NS
C12:0	3.77	3.42	0.032	NS
C14:0	13.38	12.90	0.601	NS
C15:0	1.95	2.05	0.028	NS
C16:0	40.03	35.81	0.020	<0.05
C16:1	2.04	2.03	0.045	NS
C17:0	0.97	0.91	0.034	NS
C17:1	0.45	0.48	0.056	NS
C18:0	8.73	11.88	0.036	<0.05
C18:1 *trans-11*	0.85	1.30	0.030	<0.01
C18:1 *cis-9*	16.42	23.33	0.067	<0.01
C18:2 *cis-9, cis-12*	1.84	2.24	0.035	<0.01
C18:2 *trans-10, cis-12*	0.33	0.42	0.010	<0.05
C18:2 *cis-9, trans-11*	0.68	0.81	0.020	<0.01
C18:3 *cis-9, cis-12, cis-15*	0.25	0.28	0.010	NS
C20:0	0.39	0.30	0.044	NS
C20:1n9	0.45	0.43	0.065	NS

CTR group: ten cheese wheels after 60 days of ripening obtained from the milk of dairy cows fed concentrate without olive cake supplements. OC group: ten cheese wheels after 60 days of ripening obtained from the milk of dairy cows fed concentrate with olive cake supplements. Each sample of cheese was analyzed in triplicate. SEM: standard error of the mean. ^§^ g/100 g, considering 100 g the summation of the areas of all fatty acid methyl esters identified. C4:0 = Butyric acid. C6:0 = Caproic acid. C8:0 = Caprylic acid. C10:0 = Capric acid. C12:0 = Lauric acid. C14:0 = Myristic acid. C15:0 = Pentadecanoic acid. C16:0 = Palmitic acid. C16:1 = Palmitoleic acid. C17:0 = Heptadecanoic acid. C17:1 = Heptadecenoic acid. C18:0 = Stearic acid. NS: Not Significant.

**Table 8:** Effect of olive cake supplement on cheese fatty acid classes (g/100 g) ^§^ and nutritional indices after 60 days of ripening.

No. of Observations	CTR	OC	SEM	*p*-Value
	10	10		
SFA	76.69	73.54	0.930	<0.05
MUFA	20.21	27.57	0.435	<0.01
PUFA	3.10	3.75	0.065	<0.01
CLA	1.01	1.23	0.030	<0.01
UFA/SFA	0.30	0.43	0.011	<0.01
Atherogenic index	4.16	2.90	0.456	<0.01
Thrombogenic index	5.02	3.69	0.392	<0.01
HH	0.35	0.53	0.01	<0.01

CTR group: ten cheese wheels after 60 days of ripening obtained from the milk of dairy cows fed concentrate without olive cake supplements. OC group: ten cheese wheels after 60 days of ripening obtained from the milk of dairy cows fed concentrate with olive cake supplements. HH: hypocholesterolemic/hypercholesterolemic ratio. Each sample of cheese was analyzed in triplicate. SEM: standard error of the mean. ^§^ g/100 g, considering 100 g the summation of the areas of all fatty acid methyl esters identified.
